# Residual SYNTAX score and optical coherence tomography–derived thin-cap fibroatheroma in patients with ST-elevation myocardial infarction undergoing percutaneous coronary intervention: an exploratory descriptive pilot study

**DOI:** 10.1186/s44348-026-00104-6

**Published:** 2026-10-26

**Authors:** Meharunnisha Syed, Uma Devi Karuru, Sunitha Arumulla, Neusha Doddi, Pradeep Rongali, Rama Kumari Nuthalapati

**Affiliations:** Department of Cardiology, Nizam’s Institute of Medical Sciences, Hyderabad, India

**Keywords:** ST-elevation myocardial infarction, Optical coherence tomography, Thin-cap fibroatheroma, Residual SYNTAX score

## Abstract

**Background:**

ST-elevation myocardial infarction (STEMI) remains a leading cause of global morbidity and mortality. While percutaneous coronary intervention (PCI) is the definitive reperfusion strategy, residual disease burden and plaque vulnerability both influence clinical recovery. The residual SYNTAX score (rSS) quantifies untreated macroscopic coronary artery disease, whereas optical coherence tomography (OCT) resolves microscopic plaque architecture, notably thin-cap fibroatheroma (TCFA). This pilot study explored the descriptive characteristics and short-term outcomes associated with combining rSS and OCT-derived TCFA in a highly selected STEMI cohort.

**Methods:**

This retrospective observational pilot study included 30 patients with STEMI who underwent primary PCI and culprit lesion OCT evaluation at a tertiary care center. Patients were stratified into four groups according to the presence of culprit TCFA and the post-PCI rSS threshold (≤ 4 vs. > 4): group 0 (TCFA absent, rSS > 4), group 1 (TCFA absent, rSS ≤ 4), group 2 (TCFA present, rSS > 4), and group 3 (TCFA present, rSS ≤ 4). The primary clinical endpoint was major adverse cardiac events (MACE)—a composite of all-cause death, recurrent myocardial infarction, target lesion revascularization, heart failure hospitalisation, and ischemic stroke—assessed over 3 months.

**Results:**

The cohort (mean age, 54.5 ± 10.1 years; 83.3% male, 40% with diabetes mellitus) had a 53.3% prevalence (16 of 30) of culprit lesion TCFA. Complete or reasonable revascularization (rSS ≤ 4) was achieved in 12 patients (40.0%). The overall cumulative 3-month MACE rate was 30.0% (9 of 30). MACE occurred in 1 of 7 patients (14.3%) in group 0, 1 of 7 (14.3%) in group 1, 4 of 11 (36.4%) in group 2, and 3 of 5 (60.0%) in group 3 (Fisher exact test, *P* = 0.23; log-rank *P* = 0.38). Patients with TCFA accounted for 7 of 9 clinical events (77.8%). Given the small subgroup sizes (*n* < 12), multivariable predictive risk modeling was not performed.

**Conclusions:**

In this exploratory pilot cohort of STEMI patients undergoing PCI, the presence of OCT-derived TCFA was descriptively associated with higher short-term adverse event rates across both complete and incomplete revascularization strata. Given the small sample size and clear indication bias, these findings are strictly hypothesis-generating and support the need for adequately powered prospective registries to evaluate dual anatomical-biological risk stratification.

## Background

ST-elevation myocardial infarction (STEMI) remains a major global public health challenge, contributing significantly to cardiovascular morbidity and mortality. Its incidence varies across geographic regions and demographic groups, heavily influenced by factors such as age, sex, lifestyle, and access to timely medical care [1]. The underlying pathophysiology of STEMI involves the rupture of a vulnerable atherosclerotic plaque, typically resulting in thrombus formation and acute occlusion of a coronary artery, leading to myocardial ischemia and infarction.

The residual SYNTAX score (rSS), a modified version of the original SYNTAX score, quantifies the burden of residual coronary artery disease after percutaneous coronary intervention (PCI). A higher rSS has been consistently associated with adverse cardiovascular outcomes, including increased rates of mortality, myocardial infarction, and repeat revascularization [2, 3]. In high-risk clinical subsets, such as acute coronary syndromes and cardiogenic shock, elevated residual scores further heighten adverse procedural risk [4].

Optical coherence tomography (OCT) is an intravascular imaging modality that provides high-resolution, cross-sectional images of coronary arteries. OCT enables detailed visualization of plaque components and can reliably identify thin-cap fibroatheroma (TCFA), a morphological feature strongly associated with plaque vulnerability, restenosis and rupture [5, 6]. Compared to intravascular ultrasound, OCT provides superior near-field axial resolution, which is critical for measuring thin fibrous caps in vivo [7].

Understanding the interplay between plaque characteristics, especially TCFA, and residual coronary disease burden, as quantified by rSS, could significantly improve post-PCI risk stratification, complementing classical anatomical scoring with in vivo microstructural assessment [8, 9]. This study aims to evaluate the prognostic significance of TCFA identified by OCT in conjunction with rSS in predicting major adverse cardiovascular events (MACE) in patients with STEMI undergoing PCI.

## Methods

### Ethics statement

The study was approved by the Institutional Ethics Committee of Nizam’s Institute of Medical Sciences (No. EC/NIMS/3255/2023). Written informed consent was obtained from all patients prior to their inclusion. All research was conducted in accordance with the principles of the Declaration of Helsinki.

### Study design and setting

This retrospective observational study was conducted at our center from April 2023 to March 2024. The study included patients aged 18 years or older admitted to the cardiology inpatient department with STEMI who underwent OCT for culprit lesion assessment. Exclusion criteria included cardiogenic shock, severe kidney dysfunction, chronic total occlusions, extremely tortuous arteries, left main lesions, and contrast allergies.

### Patient enrollment and diagnosis

**Fig. 1:**
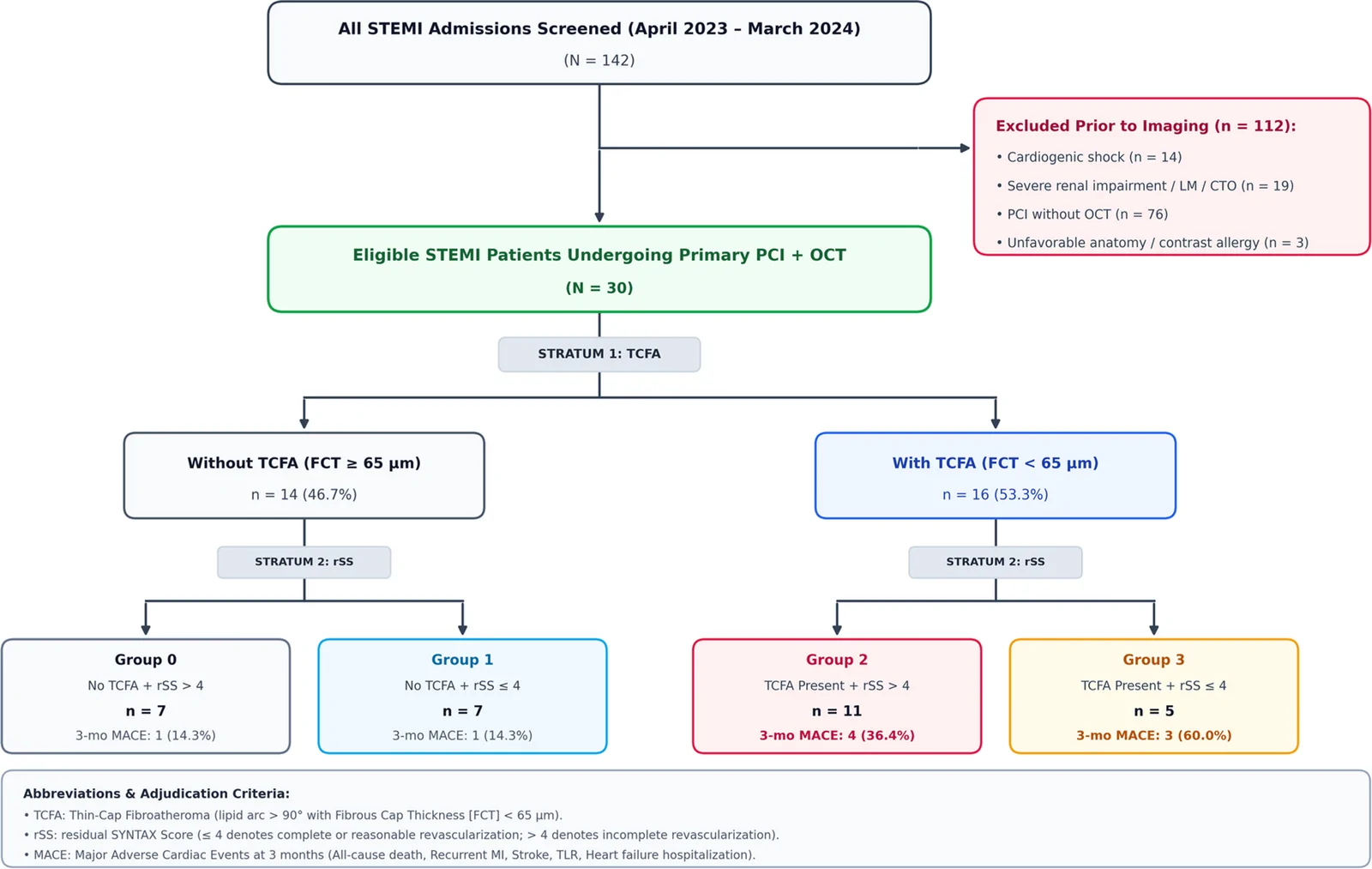
Flow diagram of patient screening, selection, and anatomical-biological group stratification. The diagram shows all acute ST-elevation myocardial infarction (STEMI) presentations screened during the study period, documented exclusions, and final cohort allocation into the four study groups based on culprit lesion thin-cap fibroatheroma (TCFA) and residual SYNTAX score (rSS; threshold, ≤ 4 vs. > 4). PCI, percutaneous coronary intervention; OCT, optical coherence tomography; LM, left main (coronary artery); CTO, chronic total occlusion; FCT, fibrous cap thickness; MI, myocardial infarction, TLR, target lesion revascularization

Patients presenting with STEMI were enrolled based on the presence of persistent, typical chest pain; electrocardiographic evidence of ST-segment elevation > 0.1 mV in at least two contiguous leads or new-onset left bundle branch block; and elevated cardiac biomarkers. The culprit lesion was identified via coronary angiography, ST-segment changes, and/or regional wall motion abnormalities on 2D echocardiography. Patients were stratified into four distinct groups (Fig. 1): (1) group 0, TCFA absent and rSS > 4; (2) group 1, TCFA absent and rSS ≤ 4; (3) group 2, TCFA present and rSS > 4; and (4) group 3, TCFA present and rSS ≤ 4.

### OCT acquisition

OCT imaging was performed using the C7-XR/ILUMIEN system with the Dragonfly catheter (Abbott). Automated pullback was conducted while displacing blood with continuous contrast injection.

### TCFA definition and image analysis

OCT pullbacks were analyzed offline using dedicated software by two independent cardiologists blinded to clinical outcomes. A TCFA was defined as a lipid-rich plaque with a maximum lipid arc > 90° and a fibrous cap thickness < 65 µm at the thinnest section, in accordance with consensus standards [5].

### Residual SYNTAX score stratification

**Fig. 2:**
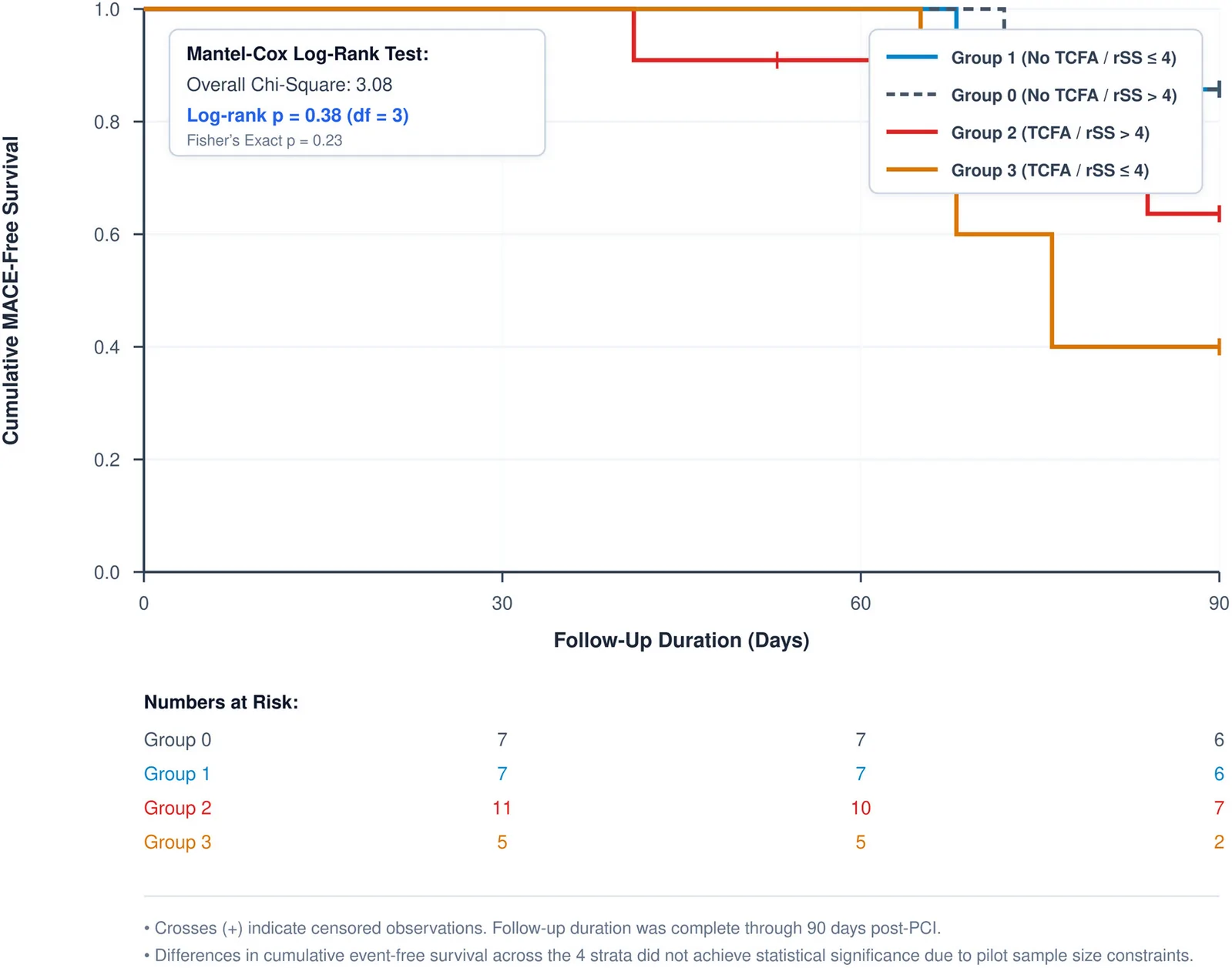
Cumulative Kaplan–Meier major adverse cardiac events (MACE)-free survival curves stratified across the four anatomical-biological study groups over the 3-month follow-up. Step curves depict event timing across group 0 (no TCFA, rSS > 4), group 1 (no TCFA, rSS ≤ 4), group 2 (TCFA present, rSS > 4), and group 3 (TCFA present, rSS ≤ 4). Censored patients are denoted with vertical tick marks (+). Numbers at risk are displayed below the time axis at 30-day intervals. Survival differences across strata were evaluated using the Mantel-Cox log-rank test (*P* = 0.38)

The residual SYNTAX score was determined post-PCI on completion angiograms. Patients were stratified using an rSS threshold of ≤ 4 versus > 4, chosen because an rSS ≤ 4 has been established as a clinically validated benchmark for reasonably complete revascularization with low long-term adverse ischemic risk [3, 10]. The primary endpoint was the cumulative incidence of MACE (all-cause mortality, recurrent MI, ischemic stroke, target vessel revascularization, and heart failure) assessed at 1, 2, and 3 months (Fig. 2). All events were adjudicated by at least two independent cardiologists.

### Statistical analysis

Continuous variables are expressed as the mean ± standard deviation and were compared across the four study strata using one-way analysis of variance or Kruskal–Wallis tests as appropriate for their distributions. Categorical variables are presented as absolute counts and percentages and were analyzed using the Fisher exact test because of sparse cell frequencies (*n* < 5). Cumulative event-free survival across the groups was estimated using the Kaplan–Meier method and compared via the log-rank (Mantel-Cox) test. Because of the pilot sample size (*n* = 30) and limited total endpoint count (*n* = 9), multivariable Cox proportional hazards regression was intentionally omitted to prevent severe mathematical overfitting. All statistical tests were two-tailed, and analyses were conducted using IBM SPSS ver. 26.0 (IBM Corp).

## Results

### Baseline clinical and angiographic characteristics

From April 2023 to March 2024, a total of 142 patients presenting with STEMI were screened, of whom 30 met all eligibility criteria, underwent culprit lesion OCT evaluation, and were allocated into the four study groups (Fig. 1). The overall cohort had a mean age of 54.5 ± 10.1 years and a predominance of male patients (*n* = 25, 83.3%). Common risk factors included hypertension (53.3%), diabetes mellitus (40.0%), dyslipidemia (36.7%), and smoking (33.3%). Baseline clinical presentation most frequently involved anterior wall myocardial infarction (46.7%). Mean left ventricular ejection fraction at baseline was 46.5% ± 8.3%. Detailed baseline clinical, angiographic, and procedural characteristics stratified by group are presented in Table 1.

**Table 1:** Baseline demographics and optical coherence tomography characteristics

Variable	Total cohort (*n* = 30)	Group 0 (*n* = 7)	Group 1 (*n* = 7)	Group 2 (*n* = 11)	Group 3 (*n* = 5)	*P*-value
Age (yr)	54.5 ± 10.1	54.9 ± 12.9	51.1 ± 13.4	57.5 ± 7.0	51.8 ± 7.7	0.62
Male sex	25 (83.3)	6 (85.7)	6 (85.7)	9 (81.8)	4 (80.0)	0.98
Hypertension	16 (53.3)	4 (57.1)	3 (42.9)	6 (54.5)	3 (60.0)	0.91
Diabetes mellitus	12 (40.0)	2 (28.6)	2 (28.6)	6 (54.5)	2 (40.0)	0.64
Dyslipidemia	11 (36.7)	2 (28.6)	3 (42.9)	4 (36.4)	2 (40.0)	0.95
Current smoker	10 (33.3)	2 (28.6)	2 (28.6)	4 (36.4)	2 (40.0)	0.95
Anterior STEMI	14 (46.7)	3 (42.9)	3 (42.9)	5 (45.5)	3 (60.0)	0.92
Baseline LVEF (%)	46.5 ± 8.3	47.1 ± 7.9	48.0 ± 9.1	45.1 ± 8.4	46.2 ± 8.8	0.90
Multivessel disease	15 (50.0)	5 (71.4)	1 (14.3)	8 (72.7)	1 (20.0)	0.02
Culprit vessel						0.88
LAD	14 (46.7)	3 (42.9)	3 (42.9)	5 (45.5)	3 (60.0)	
RCA	11 (36.7)	3 (42.9)	3 (42.9)	4 (36.4)	1 (20.0)	
LCx	5 (16.7)	1 (14.3)	1 (14.3)	2 (18.2)	1 (20.0)	
Post-PCI TIMI 3 flow	29 (96.7)	7 (100)	7 (100)	10 (90.9)	5 (100)	0.77
Stent length (mm)	28.8 ± 6.6	28.1 ± 6.2	26.4 ± 5.8	31.2 ± 7.1	27.8 ± 6.5	0.48
Fibrous cap thickness (µm)	76.1 ± 20.1	89.6 ± 10.8	90.8 ± 13.2	55.0 ± 2.2	54.4 ± 5.4	< 0.001

Values are presented as mean ± standard deviation or number (%), unless otherwise specified. Group 0: no TCFA, rSS > 4; group 1: no TCFA, rSS ≤ 4; group 2: TCFA present, rSS > 4; group 3: TCFA present, rSS ≤ 4

*STEMI* ST-elevation myocardial infarction, *LVEF* left ventricular ejection fraction, *LAD* left anterior descending coronary artery, *RCA* right coronary artery, *LCx* left circumflex coronary artery, *PCI* percutaneous coronary intervention, *TIMI* Thrombolysis in Myocardial Infarction, *TCFA* thin-cap fibroatheroma, *rSS* residual SYNTAX score

### OCT morphological observations

OCT identified TCFA in 16 culprit lesions (53.3%, categorized as groups 2 and 3). The mean fibrous cap thickness in patients with TCFA was 55.0 ± 2.2 μm in group 2 and 54.4 ± 5.4 μm in group 3, compared to 89.6 ± 10.8 μm in group 0 and 90.8 ± 13.2 μm in group 1 (*P* < 0.001).

### Clinical outcomes at 3-month follow-up

During the 3-month follow-up, MACE occurred in 9 of 30 patients (30.0%). The specific breakdown of cumulative adverse events at 3 months is summarized in Table 2: heart failure in three patients (10.0%), ischemic stroke in two (6.7%), target lesion revascularization in two (6.7%), recurrent MI in one (3.3%), and all-cause death in one (3.3%).

**Table 2:** Cumulative 3-month MACE across the study groups

Outcome	Total cohort (*n* = 30)	Group 0 (*n* = 7)	Group 1 (*n* = 7)	Group 2 (*n* = 11)	Group 3 (*n* = 5)
Total MACE	9 (30.0)	1 (14.3)	1 (14.3)	4 (36.4)	3 (60.0)
Death	1 (3.3)	0 (0)	0 (0)	0 (0)	1 (20.0)
Recurrent MI	1 (3.3)	0 (0)	0 (0)	1 (9.1)	0 (0)
Ischemic stroke	2 (6.7)	0 (0)	1 (14.3)	1 (9.1)	0 (0)
Target lesion revascularization	2 (6.7)	0 (0)	0 (0)	0 (0)	2 (40.0)
Heart failure hospitalization	3 (10.0)	1 (14.3)	0 (0)	2 (18.2)	0 (0)

Values are presented as number (%). Group 0: no TCFA, rSS > 4; group 1: no TCFA, rSS ≤ 4; group 2: TCFA present, rSS > 4; group 3: TCFA present, rSS ≤ 4. Fisher exact test across the four groups yielded P = 0.23; log-rank test for cumulative survival yielded *P* = 0.38

*MACE* major adverse cardiac events, *MI* myocardial infarction, *TCFA* thin-cap fibroatheroma, *rSS* residual SYNTAX score

When stratified by biological plaque characteristics alone, patients with TCFA (groups 2 and 3 combined) exhibited a descriptively higher event frequency (7 of 16, 43.8%) than those without TCFA (groups 0 and 1; 2 of 14, 14.3%). Across the four discrete strata, the incidence was 1 of 7 (14.3%) in group 0, 1 of 7 (14.3%) in group 1, 4 of 11 (36.4%) in group 2, and 3 of 5 (60.0%) in group 3.

When evaluated across the stratified subgroups, cumulative MACE occurred in 1 of 7 (14.3%) in group 0, 1 of 7 (14.3%) in group 1, 4 of 11 (36.4%) in group 2, and 3 of 5 (60.0%) in group 3 (Fisher exact test, *P* = 0.23). Patients with TCFA (groups 2 and 3 pooled) accounted for 7 of 9 documented primary events (77.8%). Kaplan–Meier survival curves depicting cumulative MACE-free survival across the four anatomical-biological strata showed no statistically significant separation over the 3-month observation window (Mantel-Cox log-rank ${\chi }^{2}$=3.08, *P* = 0.38) (Fig. 2).

## Discussion

This retrospective pilot study explored the observational relationship between OCT-derived plaque vulnerability and the anatomical burden of residual coronary disease in STEMI patients undergoing PCI. The primary descriptive observation in this small cohort was that patients with TCFA experienced the majority of short-term adverse events across both complete and incomplete revascularization subsets. While these observations are hypothesis-generating and constrained by small subgroup sizes and clear selection bias, they conceptually align with the premise that comprehensive post-STEMI risk stratification requires assessing both the macroscopic anatomical disease burden and the microscopic biological instability of the coronary vasculature.

### The role of incomplete revascularization and the rSS threshold

Quantifying incomplete revascularization using the rSS is well established in contemporary interventional cardiology. Farooq et al. [2], in a landmark substudy of the SYNTAX trial, demonstrated that a higher rSS is a powerful independent predictor of mortality and adverse ischemic outcomes. Similarly, Généreux et al. [10] validated the prognostic utility of rSS across multiple large cohorts, including the ACUITY trial, confirming that untreated coronary lesions serve as active substrates for recurrent ischemia and heart failure.

While large-scale clinical trials frequently evaluate rSS across tertiles (0, 1–8, > 8), our pilot study applied a dichotomous threshold of rSS ≤ 4 versus > 4. This threshold was selected based on pooled analyses from the ACUITY and SYNTAX trials [10], which established that an rSS ≤ 4 represents "reasonable revascularization" and has a prognosis comparable to that associated with an rSS of 0, whereas an rSS > 4 is associated with significantly heightened ischemic risk. Residual anatomical stenosis in a pro-thrombotic and inflammatory post-STEMI environment provides a clear physiological substrate for recurrent ischemic events.

### OCT and the biological assessment of plaque vulnerability

While rSS maps macroscopic anatomical disease, it cannot evaluate intraplaque composition or biological stability. Plaque vulnerability is morphologically characterized by a large lipid pool, macrophage infiltration, and a thin fibrous cap. As detailed by Prati et al. [5], OCT provides the requisite high-resolution cross-sectional imaging (10–20 μm) to measure fibrous cap thickness and identify TCFA in vivo. Furthermore, underlying TCFA and large lipid cores at stent margins have been directly linked to incomplete neointimal strut coverage and adverse vascular healing [11].

In our descriptive cohort, TCFA was identified in 53.3% of culprit lesions, with affected patients exhibiting substantially thinner fibrous caps (mean, 54–55 μm) compared with those without TCFA (mean, approximately 90 μm). The clinical implications of these vulnerable features were validated in the prospective CLIMA study [12], which confirmed that OCT-defined high-risk plaque characteristics, specifically TCFA, correlate with adverse clinical outcomes.

### Anatomical and biological risk profiling: prior evidence and novelty

The concept of integrating plaque morphology with residual anatomical disease has been evaluated in larger cohorts. Zhao et al. [13] investigated 274 STEMI patients undergoing primary PCI and demonstrated that combining OCT-defined plaque characteristics with the residual SYNTAX score substantially enhanced post-PCI risk stratification. Similarly, Wang et al. [14] examined OCT culprit plaque morphology alongside rSS tertiles, confirming that patients with high-risk lipid plaques and elevated residual scores experienced the poorest clinical outcomes.

Our descriptive pilot adds to this existing literature by exploring these concepts within an Indian tertiary care STEMI population characterized by premature coronary artery disease and a high baseline prevalence of diabetes mellitus (40%). Furthermore, we evaluated the feasibility of combining a simplified, clinically practical binary cutoff (rSS ≤ 4 vs. > 4) with culprit lesion TCFA detection during emergency PCI.

In our cohort, cumulative 3-month MACE occurred in 14.3% of group 0 (no TCFA, rSS > 4), 14.3% of group 1 (no TCFA, rSS ≤ 4), 36.4% of group 2 (TCFA, rSS > 4), and 60.0% of group 3 (TCFA, rSS ≤ 4). The higher observed event rate in group 3 compared to group 2 must be interpreted with caution. With a denominator of only five patients in group 3, individual clinical events exert a disproportionately large effect on the event rate (each event represents a 20% shift). Two of the three events in group 3 were repeat target lesion revascularizations. Rather than reflecting true biological resistance to complete revascularization, this numerical disparity highlights the extreme sampling variability inherent to very small subgroup strata. When aggregated by biological plaque vulnerability alone, patients presenting with TCFA (groups 2 and 3) exhibited an overall MACE rate of 43.8% compared to 14.3% in patients without TCFA (groups 0 and 1), suggesting that early short-term events in this selected cohort were predominantly driven by biological plaque instability.

### Limitations and methodological considerations

This study has several important limitations that must be considered when interpreting the findings.

#### Selection bias and atypical clinical presentation

The observed 3-month MACE rate of 30% is exceptionally elevated for a relatively young STEMI cohort (mean age, 54.5 years). Additionally, the ischemic stroke rate (*n* = 2, 6.7%) exceeded the recurrent MI rate (*n* = 1, 3.3%), which is atypical for standard acute coronary syndrome registries. This distribution strongly indicates procedural indication bias; OCT was not deployed systematically across all-comer STEMI admissions but was preferentially utilized in anatomically or clinically complex presentations. Consequently, this cohort represents a highly skewed tertiary subgroup, severely limiting external validity and generalizability to routine clinical practice.

#### Culprit lesion–only assessment

OCT interrogation was restricted exclusively to the acute culprit lesion. Landmark intravascular imaging studies, such as the PROSPECT trial [15], demonstrated that nonculprit vulnerable plaques are major drivers of late recurrent cardiovascular events. While the residual SYNTAX score quantifies the angiographic burden of untreated nonculprit lesions, the biological vulnerability (e.g., presence of TCFA) of nonculprit plaques was not imaged in our cohort, leaving a key facet of systemic vascular vulnerability unmeasured**.**

#### Sample size and statistical constraints

With only 30 patients distributed across four small subgroups (ranging from 5 to 11 patients per stratum), the study is severely underpowered. With a total of nine primary endpoints across the cohort, multivariable Cox proportional hazards modeling or formal predictive risk adjustment could not be performed without severe overfitting. The data remain purely exploratory, descriptive, and hypothesis-generating.

#### Single-center retrospective design

The single-center design and short 3-month follow-up window restrict our observations to immediate post-PCI events. Longer-term follow-up is necessary to assess whether the prognostic impact of untreated nonculprit disease burden (rSS) compounds over time relative to index plaque morphology.

## Conclusions

In this exploratory pilot cohort of STEMI patients undergoing PCI, the presence of OCT-derived thin-cap fibroatheroma was descriptively associated with a higher incidence of short-term adverse cardiac events across both complete and incomplete revascularization strata. However, given the small sample size, clear selection bias resulting in atypical clinical event rates, and the lack of statistical power for formal multivariable modeling, these findings are strictly hypothesis-generating. The observed data support the biological rationale for integrating intravascular plaque characterization with macroscopic residual disease scoring, but larger, adequately powered prospective multicenter registries are required to establish whether this dual anatomical-biological stratification provides independent prognostic value in routine post-STEMI care.

## Data Availability

No datasets were generated or analysed during the current study.

## Abbreviations

MACE: Major adverse cardiovascular events
MI: Myocardial infarction
OCT: Optical coherence tomography
PCI: Percutaneous coronary intervention
rSS: Residual SYNTAX score
STEMI: ST-elevation myocardial infarction
TCFA: Thin-cap fibroatheroma
